# Smoking behavior associated upregulation of SERPINB12 promotes proliferation and metastasis via activating WNT signaling in NSCLC

**DOI:** 10.1186/s13019-024-02625-x

**Published:** 2024-03-19

**Authors:** Hong-Zhen Zheng, Xiang Miao, Jing Chang, Hai Zhou, Jing-Jian Zhang, Hui-Min Mo, Qin Jia

**Affiliations:** Department of Respiratory Medicine, Shidong Hospital, Yangpu District, 999 Shiguang Road, Yangpu District, Shanghai, 200438 P.R. China

**Keywords:** SERPINB12, NSCLC, Wnt signaling, Proliferation, Metastasis

## Abstract

**Background:**

Non-small cell lung cancer (NSCLC) is the leading cause of morality among all malignant tumors. Smoking is one of the most important causes of NSCLC, which contributes not only to the initiation of NSCLC but also to its progression. The identification of specific biomarkers associated with smoking will promote diagnosis and treatment.

**Methods:**

Data mining was used to identify the smoking associated gene SERPINB12. CCK8 assays, colony formation assays, a mouse xenograft model and transwell assays were performed to measure the biological functions of SERPINB12 in NSCLC. GSEA, luciferase reporter assays and immunofluorescence were conducted to explore the potential molecular mechanisms of SERPINB12 in NSCLC.

**Results:**

In this study, by data mining the TCGA database, we found that SERPINB12 was greatly upregulated in NSCLC patients with cigarette consumption behavior, while the expression level was positively correlated with disease grade and poor prognosis. SERPINB12 is a kind of serpin peptidase inhibitor, but its function in malignant tumors remains largely unknown. Functionally, knockdown of SERPINB12 observably inhibited the proliferation and metastasis of NSCLC cells in vitro and in vivo. Moreover, downregulation of SERPINB12 attenuated Wnt signaling by inhibiting the nuclear translocation of β-catenin, which explained the molecular mechanism underlying tumor progression.

**Conclusions:**

In conclusion, SERPINB12 functions as a tumorigenesis factor, which could be a promising biomarker for NSCLC patients with smoking behavior, as well as a therapeutic target.

**Supplementary Information:**

The online version contains supplementary material available at 10.1186/s13019-024-02625-x.

## Background

Smoking is still believed to be one of the most important reasons for the initiation and progression of malignant cancer, especially for Non-small cell lung cancer (NSCLC) [1–3]. The overall survival of NSCLC was less than 30% [4]. Smoking is one of the most important causes of NSCLC. Smoking-associated lung cancer is recognized as the main cause of oncogenic mutations compared with nonsmoking-associated lung cancer [5]. The long latency of 30 years between tobacco exposure and lung cancer development most likely explains the delay between lung cancer incidence rates reflecting the danger of smoking. However, until now, accurately identifying the aberrant genes associated with smoking has been a major problem. In fact, the biomarkers for smokers suffering from NSCLC are still far from satisfactory. Thus, we aimed to identify biomarkers for smokers to prevent the morbidity of NSCLC.

In this study, we analyzed the differentially expressed genes between smokers and nonsmokers from The Cancer Genome Atlas (TCGA) data and found that SERPINB12 was extremely elevated in smokers. Serpin family B member 12 (SERPINB12) is a member of the SERPINB family, which is related to the innate immune system [6, 7]. Serine protease inhibitors (serpins) are a superfamily containing more than 1500 serpin genes among all organisms, which are mainly clustered on chromosome 18 (18q21) [8]. For human beings, there are 36 kinds of serpins. The serpins perform diverse functions, such as hormone transporters to regulate blood pressure, but the most common function is serine protease inhibitors [9–11].

SERPINB12 is a member of the SERPINB family, but its function in cancer is unknown. For other family members of SERPINB, the decreased expression of SERPINB1 was correlated with the invasion and metastasis of hepatocellular carcinoma [12]. The missense mutation of SERPINB10 was found to be associated with prostate cancer initiation and progression [13, 14]. However, there is no research on SERPINB12 in lung cancer. Herein, we first reported the function of SERPINB12 in NSCLC, which will also provide new insight into NSCLC biomarkers for smokers.

In this study, we successfully determined that SERPINB12 was significantly overexpressed in smokers suffering from NSCLC. Next, we intended to investigate the oncogenic functions of SERPINB12, such as proliferation, metastasis, and apoptosis, etc. Moreover, we aimed to clarify the molecular mechanism of SERPINB12 in NSCLC. Our study will provide a novel biomarker for smokers, which will possibly reduce the incidence of NSCLC.

## Materials and methods

### Data mining

The TCGA data of NSCLC were divided to two groups according to the parameter of smoking behavior to determine the differentially expressed genes (DEGs). The TCGA data were also used to analyze the expression profile of SERPINB12 and the correlation between its expression level and smoking behavior, cigarette consumption, and pathologic stage. The Kaplan‒Meier Plotter website (http://kmplot.com/analysis/) was used to uncover the prognostic value. The Gene set enrichment analysis (GSEA) was performed following the instructions provided by the Broad Institute.

### Real-time PCR

First, the cells were washed with PBS, and TRIzol reagent was used to extract total RNA, which was reverse transcribed to cDNA via a PrimeScript RT‒PCR kit (Takara, Japan) following the instructions provided by the manufacturer. Then, SYBR green (Bimake, USA) was utilized to perform real-time PCR with a 7500 Real-time PCR system (Applied Biosystems, USA). The calculation methodology of △△CT was adopted to confirm the relative expression of specific genes, and GAPDH was the reference gene. The primer sequences are shown in Table 1.

**Table 1:** Sequences of primers used for real-time PCR

Primer	Sequence 5’-3’
BMP2 Forward	TCATAAAACCTGCAACAGCCAACTCG
BMP2 Reverse	CACCCACAGCGATCATGTCG
CDH1 Forward	ATGAGTGTCCCCCGGTATCTTC
CDH1 Reverse	ACGAGCAGAGAATCATAAGGCG
CDH2 Forward	GTGCATGAAGGACAGCCTCT
CDH2 Reverse	GCCACTTGCCACTTTTCCTG
SLUG Forward	CCTCCATCTGACACCTCC
SLUG Reverse	CCCAGGCTCACATATTCC
SNAIL Forward	GTATCCAGAGCTGTTTGGA
SNAIL Reverse	AACATTTTCCTCCCAGGCC
VIM Forward	GATGGTGTTTGGTCGCATA
VIM Reverse	GATGGTGTTTGGTCGCATA
SERPINB12 Forward	CCAACAGGCTTTATGGAGAGC
SERPINB12 Reverse	CACTTTCAATCGTCGTGTGGTAA
GAPDH Forward	CTGGGCTACACTGAGCACC
GAPDH Reverse	AAGTGGTCGTTGAGGGCAATG

### Cell culture

Human lung cell and cancer cell lines (16-HBE, H157, H1703, H358, A549, and H1650) were maintained long-term in Shidong Hospital of Yangpu District. Dulbecco’s modified Eagle’s medium (DMEM) and Roswell Park Memorial Institute 1640 medium supplemented with 10% fetal bovine serum (FBS) were utilized for cell culture. The cells were incubated under 5% CO_2_ and 37 °C.

### CCK8 assay

A Cell Counting Kit-8 (CCK8) assay was performed to test cell viability. The cells were seeded in 96-well plates at a concentration of 3000 cells/well, and CCK-8 reagent was added at the indicated times for 5 days. Cell viability was measured with a microplate reader at 450 nm absorbance.

### Colony formation assays

The digested cells were seeded into a 6-well plate with 1000 cells per well and cultured for 14 days. Subsequently, colonies were fixed with 4% paraformaldehyde for 30 min and stained with crystal violet for another 30 min. Then, the plate was washed with flowing water to form a cell colony. Ultimately, the results were obtained by a scanner and analyzed by Image J software.

### SERPINB12 knockdown by RNA interference

Specific custom sh-RNAs or sh-scramble were synthesized by GenePharma (Shanghai, China) and cloned and inserted into the pLKO.1 plasmid for packaging as a lentivirus. Cells were infected with lentivirus in the presence of 10 mg/ml polybrene (Sigma, H9268) and screened with 2 µg/ml puromycin (Gibco, A1113802) after transfection for 48 h to obtain stable knockdown cell lines. The sh-SERPINB12-1 (sh-1) sequence was 5’- GGCTGGGTCCTTAAACAATGA-3’, and the sh-SERPINB12-2 (sh-2) sequence was 5’- GGAATTCCCAATCTGTCAGGA-3’.

### Immunohistochemistry (IHC) assay

The tissues were paraffin-embedded and cut into 4 μm sections. Then, the tissue slides were dewaxed and rehydrated with xylene and graded alcohol. Citric sodium (pH = 6.0) was used to perform heat-epitope antigen retrieval by a microwave, cooling at room temperature. H2O2 (3%) dissolved in methanol was utilized to reduce endogenous peroxidase activity for 10 min. The slides were covered with bovine serum albumin (BSA) to block the antigen at room temperature for 1 h and then incubated with primary antibodies against ki-67 (Abcam, ab15580) and β-catenin (ab32572, Abcam) at 4 °C overnight, followed by incubation with secondary antibodies at room temperature for 1 h. The DAB reagent was used to visualize the expression.

### Tumor cell migration and invasion assays

To examine the migration and invasion ability of NSCLC cells, wound healing and transwell assays were conducted. For the wound healing assay, NSCLC cells were cultured in 6-well plates and grown to 100% confluence before the wound was generated by pipette tips, followed with replacing serum-free culture medium. The images were captured at 0, 24 and 48 h. For the transwell assay, 3 × 10^4^ cells were seeded in the upper chambers (Millipore, PIEP12R48) and cultured for 24 h. The membranes were obtained from the transwell chambers and stained with 0.1% crystal violet. Then, the images were captured with a microscope. For the invasion assay, the matrix gel was precoated into the chambers before seeding with 6 × 10^4^ cells, and the other protocols followed the instructions for the transwell migration assay after culturing for 48 h.

### Western blot analysis

Total protein was extracted by IP lysis buffer supplemented with 1% protease inhibitor and phosphatase inhibitor. The protein concentration was examined by a BCA protein assay kit. The protein was subjected to SDS‒PAGE and transferred to NC membranes. The membrane was incubated with 5% milk to block the antigens for 1 h at room temperature and then covered with primary antibodies against SERPINB12 (Abcam, ab267463), β-catenin (Abcam, ab32572), laminA/C (Abcam, ab108595) and β-actin (Abcam, ab8226) at 4 °C overnight. Species-specific secondary antibodies were used to incubate the membrane. Ultimately, the results were detected and captured by an Odyssey imaging system.

### Mouse xenograft model

Male nu/nu mice aged 6 weeks were used in this study. The subcutaneous implant model was established by injecting cells subcutaneously at a concentration of 2 × 10^6^ cells. The mice were sacrificed after one month, and the tumors were obtained.

### Immunofluorescence assay

The cells were fixed with 4% paraformaldehyde for 15 min and then permeabilized with 0.1% Triton X-100 for 15 min. After washing with PBS 3 times, the cells were blocked with 2% BSA for half an hour at room temperature. Then, the cells were incubated with primary antibody at 4% overnight. The next day, the cells were washed with PBS three times and incubated with secondary antibodies and DAPI for 1 h. Ultimately, immunofluorescence staining was obtained using a Leica confocal microscope.

### Luciferase reporter assay

NSCLC cells were seeded in 96-well plates. The cells were next transfected with a mixture of TOP (TCF reporter plasmid), reporter plasmid (Wnt/β-catenin) and Renilla plasmid. The firefly and Renilla luciferase activities were examined by dual-luciferase reporter assay protocols following the manufacturer’s instructions.

### Statistical analysis

SPSS 13 and GraphPad software were utilized to conduct statistical analysis. The Kaplan‒Meier method was adopted to measure cumulative survival time. Two-tailed Student’s t test was utilized to analyze the difference between two groups. Two-way ANOVA was adopted to evaluate the significant differences among several groups. Statistical significance was exhibited as follows: *p* > 0.05 = ns, *p* < 0.05 = *, *p* < 0.01 = **, *p* < 0.001 = ***.

## Results

### SERPINB12 was significantly upregulated in NSCLC, and highly associated with smoking

**Fig. 1:**
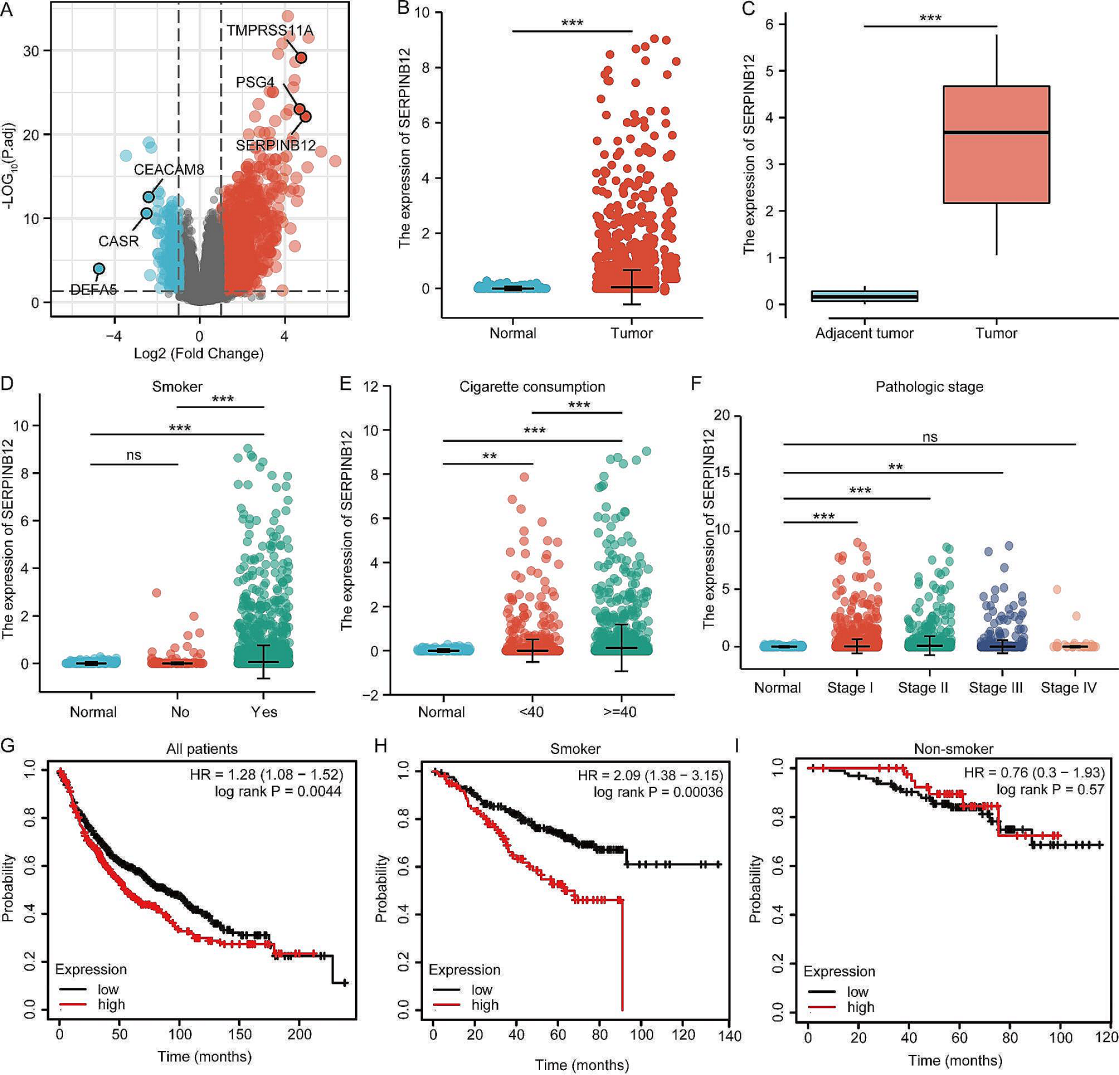
SERPINB12 was significantly upregulated in NSCLC and was highly associated with smoking. (**A**) The volcano plot shows the differentially expressed genes in smokers with NSCLC. (**B**) The expression pattern of SERPINB12 in tumor and normal tissues analyzed by TCGA data. (**C**) The expression pattern of SERPINB12 in tumor and adjacent tumor tissues analyzed by qPCR using the collected tissue. (**D**) The expression profile of SERPINB12 in normal samples and NSCLC patients with or without a habit of smoking according to TCGA data. (**E**) The expression profile of SERPINB12 according to cigarette consumption. (**F**) The expression pattern of SERPINB12 in various pathologic stages. (**G**) The overall survival of lung cancer patients with high and low expression of SERPINB12. (**H**) The overall survival of smoking patients with high and low expression of SERPINB12. (**I**) The overall survival of nonsmoking patients with high and low expression of SERPINB12. * *p* < 0.05, ** *p* < 0.01, *** *p* < 0.001

Smoking is one of the most important reasons for the initiation and progression of NSCLC, but there is still a lack of biomarkers for early diagnosis and prognosis evaluation for patients with heavy smoking history. To determine effective biomarkers for smokers suffering from NSCLC, we analyzed the differentially expressed genes (DEGs) from the TGCA database between smoking samples and nonsmoking samples. The results showed that SERPINB12 was greatly upregulated in smoking samples, which peaked our interest (Fig. 1A). Furthermore, the expression of SERPINB12 in tumors was significantly higher than that in normal samples (Fig. 1B). In addition, we also measured the expression of SERPINB12 in tumor and adjacent tumor tissues using our samples collected in Shidong Hospital of Yangpu District, which indicated that SERPINB12 was elevated in tumor tissues (Fig. 1C). Moreover, to clarify the association between SERPINB12 expression and smoking, we analyzed its expression in normal samples, nonsmoker NSCLC patients, and smokers suffering from NSCLC. The results illustrated that SERPINB12 was upregulated in smokers compared with that in nonsmokers and normal controls (Fig. 1D). In addition, the expression of SERPINB12 was positively correlated with cigarette consumption (number pack years smoked) (Fig. 1E). We also noted that SERPINB12 expression was upregulated at stage I patients, comparable to stage II and III (Fig. 1F), which means the up-regulation of SERPINB12 occurred in the early stages of tumor progression and may serve as an indicator for early screening of lung cancer. Next, we evaluated the expression of SERPINB12 in the prediction of prognosis. Intriguingly, we found that upregulated expression of SERPINB12 was associated with poor survival of NSCLC patients from the overall perspective (Fig. 1G), but higher expression of SERPINB12 was associated with worse survival in smokers (Fig. 1H) than in nonsmokers (Fig. 1I). Overall, the above results demonstrated that SERPINB12 was characteristically upregulated in NSCLC, predicting poor prognosis, making it an effective biomarker for smokers.

### SERPINB12 promoted tumor cell growth in NSCLC

**Fig. 2:**
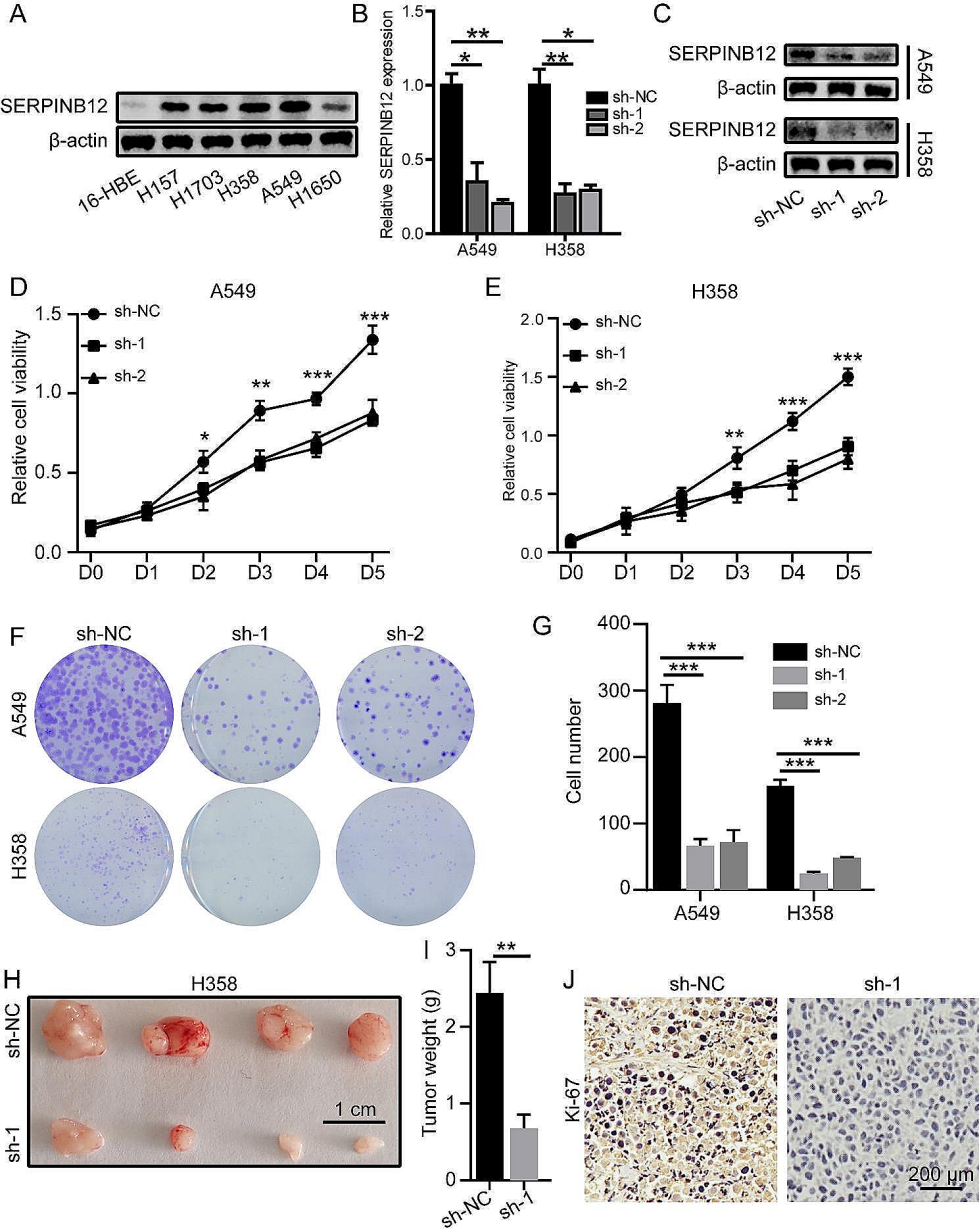
SERPINB12 promoted proliferation and tumor growth in NSCLC. (**A**) Relative protein expression of SERPINB12 in normal epithelial lung cells (16-HBE) and NSCLC cell lines, including H157, H1703, H358, A549, and H1650. (**B** and **C**) The knockdown efficacy of SERPINB12 in H358 and A549 cells was verified by qPCR and western blotting. (**D**-**E**) CCK-8 assay conducted using SERPINB12-knockdown A549 and H358 cells. (**F**-**G**) Colony formation assay conducted using SERPINB12-knockdown A549 and H358 cells. (**H**) In vivo study to evaluate tumor growth by injecting different cells whose SERPINB12 had been inhibited or not (*n* = 4 for each group, one-way ANOVA, ** *p* < 0.01). (**I**) The tumor weight in the sh-NC and sh-1 groups. (**J**) IHC staining to evaluate the expression of Ki-67 in the sh-Ctrl and sh-1 groups (scale bar: 200 μm). * *p* < 0.05, ** *p* < 0.01, *** *p* < 0.001

Next, we aimed to determine the oncogenic function of SERPINB12 in NSCLC. First, we measured the expression of SERPINB12 in normal lung epithelial cells, 16-HBE cells, and different NSCLC cells, including H157, H1703, H368, A549, and H1650 cells, through western blotting and real-time PCR. SERPINB12 expression was upregulated in cancer cells compared with that in normal lung cells (Fig. 2A and Supplementary Fig. 1). Then, we chose the A549 and H358 cell lines, whose expression levels were higher than those in other cell lines, to knockdown the expression of SERPINB12 (Fig. 2B and C), which were termed sh-NC, sh-1 and sh-2, corresponding to empty vehicle of sh-RNA treatment. After knocking down the expression of SERPINB12, the proliferation of A549 (Fig. 2D) and H358 (Fig. 2E) cells was significantly reduced. And the similar results were exhibited by colony formation of A549 and H358 cells (Fig. 2F-G). Meanwhile, H1650 cell was selected to overexpress SERPINB12 expression (Supplementary Fig. 2A). As shown in Supplementary Fig. 2B-C, H1650 cell with SERPINB12 overexpression exhibited a significantly higher proliferative ability than the control cell. Moreover, we further verified the function of SERPINB12 in tumor growth in vivo. The results illustrated that tumor growth was dramatically impaired after SERPINB12 silencing, as clarified by the tumor weight (Fig. 2H-I). In addition, the expression of Ki-67 in the SERPINB12 knockdown groups was much lower than that in the control group, which indicated that downregulation of SERPINB12 reduced tumor growth (Fig. 2J).

### Knockdown of SERPINB12 impaired the invasion and metastasis of NSCLC cells via inhibiting EMT

**Fig. 3:**
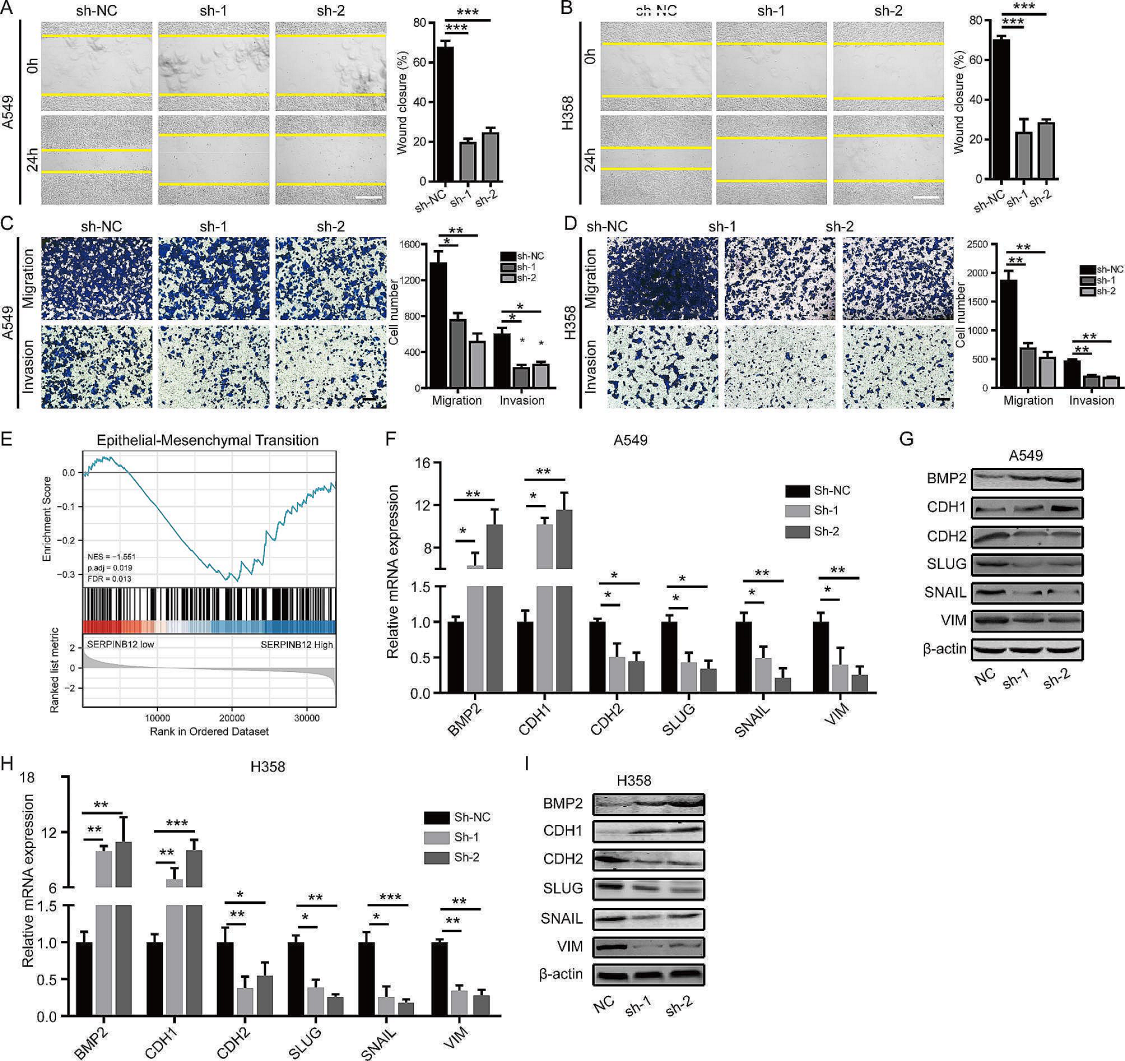
Knockdown of SERPINB12 impaired the invasion and metastasis of NSCLC cells by inhibiting EMT. (**A** and **B**) Wound healing assays conducted using SERPINB12-knockdown and control A549 and H358 cells (scale bar: 50 μm). The percentage of wound closure was calculated at 0 and 24 h. The mean ± SD from three independent experiments are shown. (**C** and **D**) Transwell assays were performed with SERPINB12-knockdown and control A549 and H358 cells to examine migration and invasion ability (scale bar: 50 μm). The numbers of cells migrating through the inserts were calculated, and the mean ± SD from three independent experiments are shown. (**E**) GSEA based on the expression profiles of samples with high and low SERPINB12 expression using the TCGA dataset. (**F** and **G**) Relative mRNA and protein expression levels of epithelial markers (BMP2 and CDH1) and mesenchymal markers (CDH2, SLUG, SNAIL, VIM) in A549 cells with or without SERPINB12 knockdown. (**H** and **I**) Relative mRNA and protein expression levels of epithelial markers (BMP2 and CDH1) and mesenchymal markers (CDH2, SLUG, SNAIL, VIM) in H358 cells with or without SERPINB12 knockdown. * *p* < 0.05, ** *p* < 0.01, *** *p* < 0.001

To examine the effect of SERPINB12 on the migration and invasion of NSCLC cells, wound healing and transwell assays were performed. As shown in Fig. 3**A**-**B**, NSCLC cells with SERPINB12 knockdown exhibited obviously weaker migration ability than control cells. Consistently, the results of the transwell assay also proved that knockdown of SERPINB12 attenuated the invasion ability of NSCLC cells in vitro (Fig. 3**C**-**D**). On the contrary, SERPINB12 overexpression significantly promotes the migration and invasion ability of H1650 cell (Supplementary Fig. 2D-E).

The process of epithelial-mesenchymal transition (EMT) results in the metastasis of malignant tumors, including NSCLC. The GSEA results showed that high expression of SERPINB12 was associated with EMT (Fig. 3**E**). Hence, we detected biomarkers of EMT after SERPINB12 knockdown. We found that knockdown of SERPINB12 resulted in significant upregulation of epithelial markers, including BMP2 and CDH1, and downregulation of mesenchymal markers (CDH2, SNAIL, SLUG and VIM) in A549 (Fig. 3**F**-**G**) and H358 cells (Fig. 3H-I).

### SERPINB12 activated Wnt/β-catenin signaling by facilitating the nuclear translocation of β-catenin

**Fig. 4:**
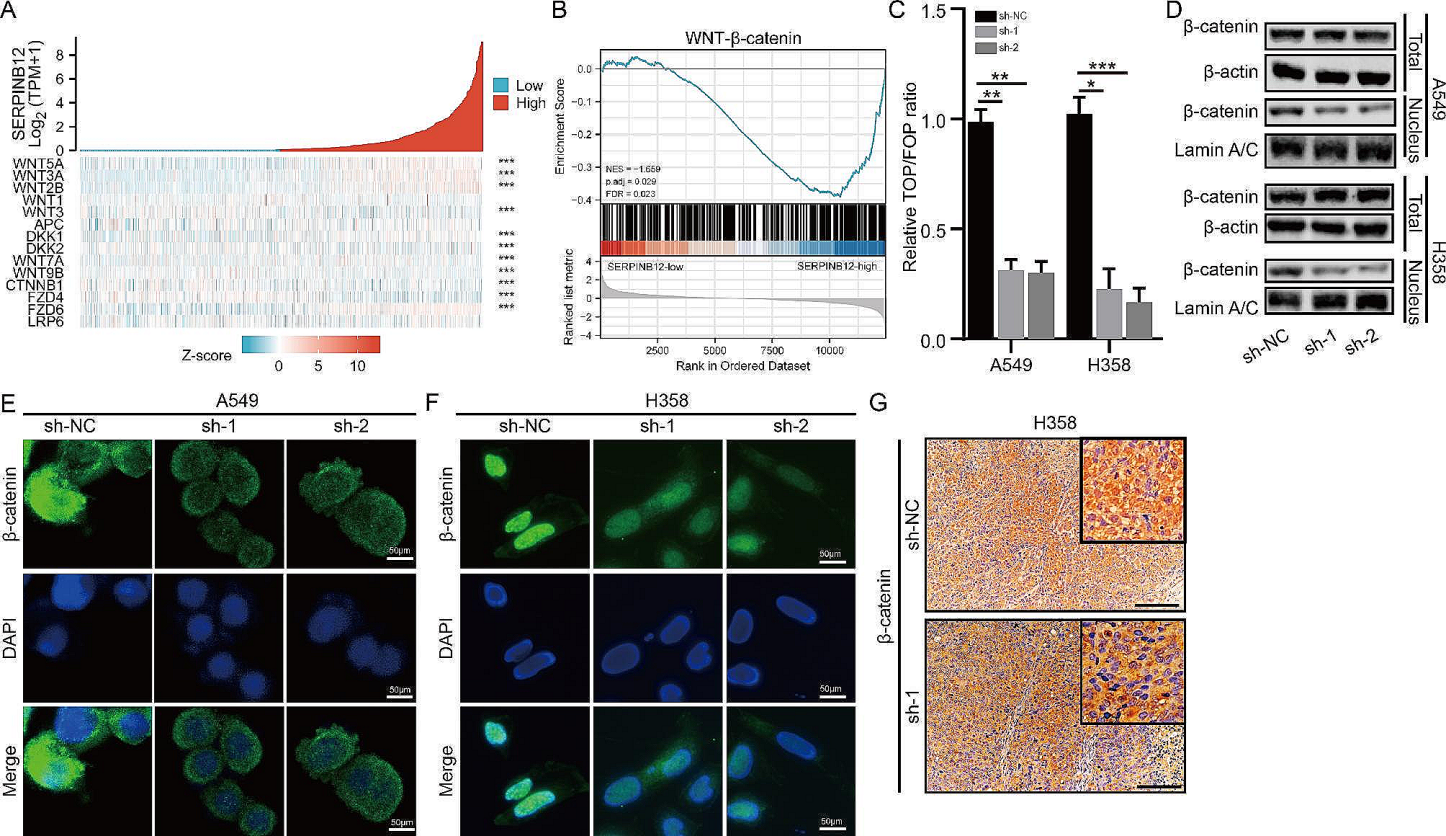
SERPINB12 activated Wnt/β-catenin signaling by facilitating the nuclear translocation of β-catenin. (**A**) The correlation between SERPINB12 and genes involved in the Wnt signaling pathway. (**B**) GSEA based on the expression profiles of samples with high and low SERPINB12 expression using the TCGA dataset. (**C**) The luciferase reporter assay of TOP/FOR was conducted in the sh-NC, sh-1, and sh-2 groups. (**D**) Nuclear and cytoplasmic expression of β-catenin in the sh-NC, sh-1, and sh-2 groups. (**E**) The translocation of β-catenin exhibited by immunofluorescence in the sh-NC, sh-1, and sh-2 groups of A549 cells (scale bar: 50 μm). (**F**) The translocation of β-catenin exhibited by immunofluorescence in the sh-NC, sh-1, and sh-2 groups of H358 cells (scale bar: 50 μm). (**G**) IHC staining of β-catenin in mouse subcutaneous neoplasia tissue formed by H358 cells treated with sh-NC or sh-1. Scale bar: 200 μm. * *p* < 0.05, ** *p* < 0.01, *** *p* < 0.001

Next, we aimed to investigate the underlying mechanism of SERPINB12 in NSCLC. To reveal the possible signaling pathway, we evaluated the correlation between SERPINB12 and typical signaling molecules. Intriguingly, we found that the expression of SERPINB12 was significantly associated with molecules of the Wnt/β-catenin signaling pathway, such as WNT5A, WNT3A, and WNT2B (Fig. 4A). Furthermore, gene set enrichment analysis (GSEA) was also performed based on SERPINB12 high expression and low expression in NSCLC, which revealed that the gene set from the SERPINB12 high-expression group was enriched in Wnt/β-catenin signaling (Fig. 4B). Hence, a TOP/FOP luciferase activity assay was performed to validate Wnt/β-catenin signaling activity. The results revealed that knockdown of SERPINB12 obviously inhibited the Wnt/β-catenin signaling pathway (Fig. 4C). Moreover, because the translocation of β-catenin from the cytoplasm to the nucleus is a feature of Wnt/β-catenin signaling pathway activation, we investigated whether SERPINB12 could enhance the translocation of β-catenin. The results indicated that knockdown of SERPINB12 expression significantly impaired the nuclear translocation of β-catenin in NSCLC cells (Fig. 4D). Relative amounts of the total and the nuclear β-catenin proteins were quantified (Supplementary Fig. 3). And SERPINB12 overexpression showcased an opposite phenomenon (Supplementary Fig. 4A). Consistently, this conclusion was also confirmed by an immunofluorescence assay, which visibly exhibited the reduced nuclear translocation of β-catenin after silencing SERPINB12 expression (Fig. 4E-F) and the increased nuclear translocation of β-catenin after SERPINB12 overexpression (Supplementary Fig. 4B) in NSCLC cells, as revealed by immunofluorescence (IF). A similar phenomenon was observed in mouse subcutaneous neoplasia tissue formed by H358 cells with SERPINB12 silencing (Fig. 4G).

## Discussion

In the present study, we found a novel biomarker, SERPINB12, to distinguish smokers and nonsmokers suffering from NSCLC. SERPINB12 was upregulated in tumor tissues compared with that in normal tissues, and it was also significantly overexpressed in smokers. Intriguingly, the expression of SERPINB12 was elevated according to the increase in cigarette consumption. SERPINB12 could also predict poor prognosis in NSCLC patients, especially smokers, which was an interesting finding in our study. Analysis of the expression profile and prognosis of SERPINB12 indicated that SERPINB12 could be a vital biomarker for NSCLC patients who have a habit of smoking.

SERPINB12 belongs to the family of serine protease inhibitors (serpins), which were identified to be involved in the innate immune system and anaphylaxis [15–17]. However, its function and molecular mechanism in cancer are unknown, and even the other serpins have not been sufficiently investigated. In this study, we found that SERPINB12 was upregulated in NSCLC cells, and knockdown of SERPINB12 expression impaired the proliferation and metastasis of NSCLC cells. SERPINB12 could facilitate the EMT process to promote tumor metastasis. Furthermore, we preliminarily explored the molecular mechanism of SERPINB12 in promoting tumor progression. Based on the bioinformatic analysis, we assumed that SERPINB12 could activate the classic Wnt signaling pathway by enhancing the translocation of β-catenin from the cytoplasm to the nucleus.

Previous studies found that Wnt signaling was strongly associated with tumor initiation and progression [18–20]. For instance, Wnt signaling promoted tumor growth, and aberrant Wnt signaling was reported to increase the cell proliferation of melanoma, liver cancer, breast cancer and other malignant tumors [21–23]. Increased Wnt signaling is also associated with metastasis and EMT in cancer [24, 25]. Two prominent branches of the Wnt signaling network are the so-called canonical WNT–β-catenin and noncanonical Wnt–planar cell polarity (PCP) pathways [26]. Wnt ligands can activate β-catenin-independent signaling pathways, which are called noncanonical Wnt signaling [27]. For canonical Wnt signaling, the translocation of β-catenin from the cytoplasm to the nucleus triggers the expression of various genes, that have been reported to participate in tumor formation [28, 29]. For instance, E-cadherin, vimentin, slug, snail, and other proteins are involved in the process of EMT [30, 31].

There are still some unraveled questions that need further exploration. For example, even though we reported the aberrant upregulation of SERPINB12 in NSCLC patients with a habit of tobacco consumption, the underlying reason for its overexpression is still unknown. We assumed that some substances from tobacco would trigger the transcriptional alteration of SERPINB12 by regulating its transcription, translation, or epigenetic change. The resolution of these unsolved problems will provide deeper insights and a better understanding of the function and application of SERPINB12 in the diagnosis of NSCLC in the future.

## Conclusions

In conclusion, our data, for the first time, identified that SERPINB12 was differentially expressed during the malignant transformation progression of NSCLC. In addition, the expression of SERPINB12 is independently associated with poor survival of NSCLC patients with a habit of smoking. Moreover, SERPINB12 promoted the invasion and metastasis of NSCLC cells. Investigations of the molecular mechanism revealed that SERPINB12 activates Wnt/β-catenin signaling to enhance proliferation and metastasis. These results indicated that SERPINB12 has the potential to be a diagnostic biomarker and therapeutic target for NSCLC patients who are long-term smokers.

### Electronic supplementary material

Below is the link to the electronic supplementary material.

Supplementary Material 1

## Data Availability

The datasets used and/or analyzed during the current study are available from the corresponding author on reasonable request.
