# Supplementary material for: Smoking behavior associated upregulation of SERPINB12 promotes proliferation and metastasis via activating WNT signaling in NSCLC
Source: J Cardiothorac Surg. 2024 Mar 19;19:141. doi: 10.1186/s13019-024-02625-x (PMC10949655; doi:10.1186/s13019-024-02625-x)
Supplement: Supplementary file 1 — Supplementary Material 1 [file 13019_2024_2625_MOESM1_ESM.docx]

**Smoking behavior associated upregulation of SERPINB12 promotes proliferation and metastasis via activating WNT signaling in NSCLC**

Hong-Zhen Zheng ^a,^ *, Xiang Miu ^a,^ *, Jing Chang ^a^, Hai Zhou ^a^, Jing-Jian Zhang ^a^, Hui-Min Mo ^a^, Qin Jia ^a^

^a^ Department of Respiratory Medicine, Shidong Hospital, Yangpu District, Shanghai, 200438, P.R. China

* These authors contributed equally to this work. **Smoking behavior associated upregulation of SERPINB12 promotes proliferation and metastasis via activating WNT signaling in NSCLC**

Hong-Zhen Zheng ^a,^ *, Xiang Miu ^a,^ *, Jing Chang ^a^, Hai Zhou ^a^, Jing-Jian Zhang ^a^, Hui-Min Mo ^a^, Qin Jia ^a^

^a^ Department of Respiratory Medicine, Shidong Hospital, Yangpu District, Shanghai, 200438, P.R. China

* These authors contributed equally to this work.


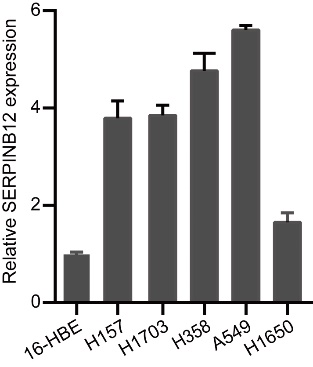


**Supplemental Fig.1** Relative mRNA expression of SERPINB12 in normal epithelial lung cells (16-HBE) and NSCLC cell lines, including H157, H1703, H358, A549, and H1650.


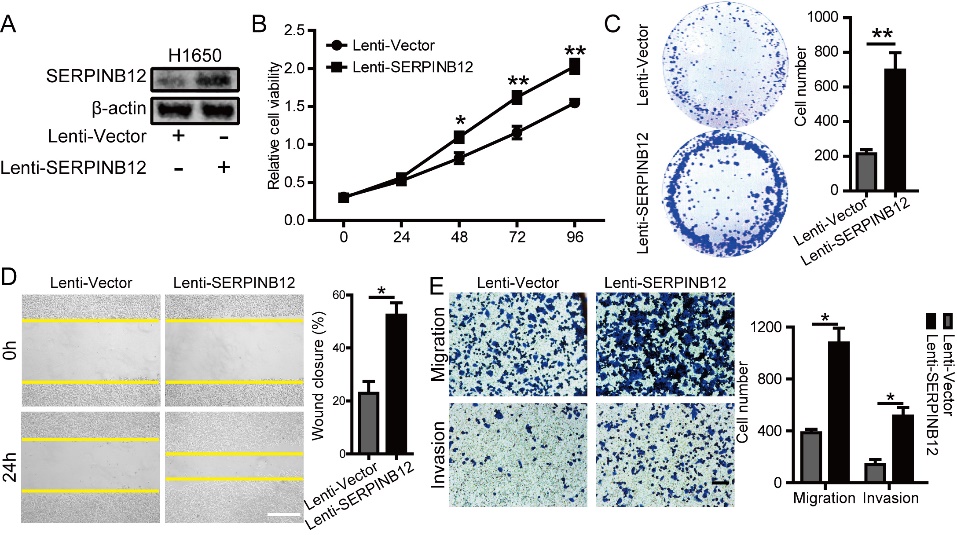


**Supplemental Fig. 2 SERPINB12 overexpression promotes NSCLC cell growth and motility.** (A) Validation of SERPINB12 overexpression in H1650 cell at the protein level. (B) Cell viabilities of H1650 cell with or without SERPINB12 overexpression were detected by CCK8 assay. (C) Colony formation assay conducted using H1650 cell stably expressing SERPINB12 or not. (D) Wound healing assay conducted using H1650 cell with or without SERPINB12 overexpression (scale bar: 50 μm). (E) Representative images and quantification of migrated and invaded H1650 cells with SERPINB12 overexpression or not tested by transwell assay. * p < 0.05, ** p < 0.01.


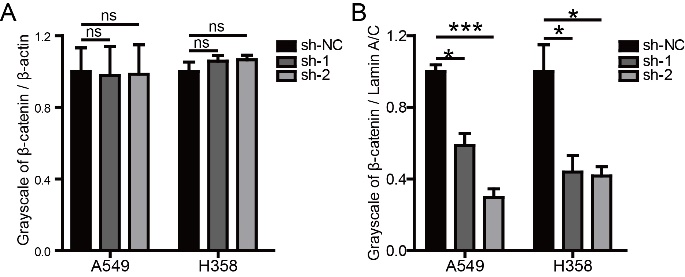


**Supplemental Fig. 3 Nuclear and cytoplasmic distribution of β-catenin.** (A) The relative β-catenin expression by normalized with the intensity of β-actin in total protein monitored by western blotting. (B) The relative β-catenin expression by normalized with the intensity of Lamin A/C in nucleus protein monitored by western blotting. ns, no significance. * p < 0.05, *** p < 0.001.


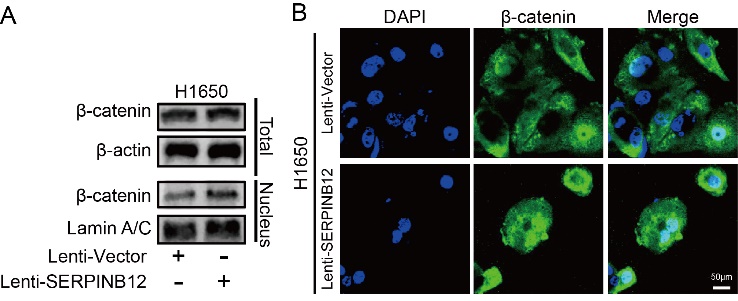


**Supplemental Fig. 4 SERPINB12 promots nuclear translocation of β-catenin.** (A) The amount of β-catenin in total protein and the amount in the nuclear were detected by western blotting. (B) The distribution of β-catenin in H1650 cell transfected with Lenti-vector or Lenti- SERPINB12 were analyzed by IF staining. Scale bars, 50 μm.
